# Saffold virus is able to productively infect primate and rodent cell lines and induces apoptosis in these cells

**DOI:** 10.1038/emi.2014.15

**Published:** 2014-02-26

**Authors:** Yishi Xu, Carla Bianca Luena Victorio, Qimei Ng, Yee Joo Tan, Kaw Bing Chua

**Affiliations:** 1 Temasek Life Sciences Laboratory, National University of Singapore, Singapore 117604, Singapore; 2 Department of Microbiology, Yong Loo Lin School of Medicine, National University Health System, National University of Singapore, Singapore 117597, Singapore; 3 Institute of Molecular and Cell Biology, Agency for Science, Technology and Research (A*STAR), Singapore 138673, Singapore

**Keywords:** apoptosis, cytopathic effect, permissible cells, Saffold virus

## Abstract

Saffold virus (SAFV), a newly discovered human cardiovirus of the *Picornaviridae* family, causes widespread infection among children, as shown by previous seroprevalence studies. To determine the host cell range of SAFV and its cytopathogenicity, eight mammalian cell lines that were available in the laboratory were screened for productive SAFV infection by a laboratory-adapted SAFV of genotype 3. Five of the cell lines (Neuro2A, CHO-K1, NIH/3T3, Vero and HEp-2) were found to be permissible. The time required for SAFV to induce complete lysis as a cytopathic effect (CPE) in these permissibly infected cells and the resultant end point virus titer differed for each cell type. HEp-2 exhibited the shortest time frame to reach full CPE compared to the others. All infected cell lines produced a high virus titer at 72 h post-infection. In addition to causing lytic cell death, SAFV also induced apoptotic cell death in host cells through both extrinsic and intrinsic pathways, although the apoptotic events in HEp-2 cells appeared to have been blocked between the early and late stages. In conclusion, laboratory-adapted SAFV is able to productively infect a number of mammalian cell lines and induce apoptosis in the infected host cells. However, apoptosis in HEp-2 cells is blocked before the end stage.

## INTRODUCTION

Saffold virus (SAFV) is a member of the *Theilovirus* species in the *Cardiovirus* genus of the family *Picornaviridae*.^1,2,3^ The virus was initially named human Theiler's-like cardiovirus due to its close phylogenetic relation to Theiler's murine encephalomyelitis virus (TMEV).^4^ Similar to other cardioviruses, SAFV is a non-enveloped single-stranded RNA virus of positive polarity. The genome of SAFV is 8050 nucleotides (nt) long and, similar to other cardioviruses, has one open reading frame with 13 cleavage sites, from which 12 separate proteins are derived: L, VP4, VP3, VP2, VP1, 2A, 2B, 2C, 3A, 3B, 3C and 3D.^5^ The 5′ untranslated region contains a type II internal ribosomal entry site that is 1040 nt long.^6^ The 3′ untranslated region, which contains the poly(A) tract, is approximately 120 nt long. Phylogenetic analysis of SAFV revealed that SAFV is genetically most closely related to Theiler-like rat virus and TMEV.^5^

Prior to the discovery of SAFV, viruses belonging to the *Cardiovirus* genus were only known to infect animals. The human origin of Vilyuisk virus, another cardiovirus, was equivocal, and the virus was suspected to be a recombinant form of human and murine cardioviruses resulting from multiple passages in the mouse brain during the process of its isolation.^7,8^ SAFV was first isolated in 1981 from a stool sample of an 8-month-old girl presenting with fever of unknown origin, but it was only characterized and reported much later, in May 2007.^5^ A year later, Abed and Boivin^9^ reported the isolation of a genotype 2 SAFV (SAFV-2) from three Canadian children exhibiting respiratory symptoms. Drexler *et al.*^10^ reported the detection of SAFV-3 from patients with acute enteritis in Germany and Brazil. Subsequently, Zoll *et al.* described the first cell-cultivatable SAFV-3 isolated from a stool sample of a 13-month-old boy in the Netherlands.^3^ In the same year, five more genotypes of SAFV were identified from stool specimens through the molecular detection of cardiovirus infection among South Asian children.^11^ Recently, 11 genotypes of SAFV were detected using consensus degenerate primers targeted against the VP1 gene region, and their respective genetic sequences were deposited in the NCBI GenBank. Furthermore, a 3-year prospective molecular epidemiological study in Denmark showed that three phylogenetically distinct lineages of SAFV-2 were introduced into the country and remained in cocirculation.^12^

The distribution of SAFV is most likely widespread, based on published data of its frequent molecular detection and the available, albeit limited, seroprevalence studies. Zoll *et al.*^3^ showed that SAFV-3 infection occurs early in life and can reach infection rates of more than 90% in children older than 24 months based on a seroprevalence study conducted in several countries in Europe, Africa and Asia. A seroprevalence study of SAFV infection conducted in the Netherlands, Finland, Cameroon and Indonesia showed that the seroprevalence of SAFV-2 was similar to that of SAFV-3.^13^ A similar trend of a high seroprevalence of SAFV-3 was also found in Malaysia, where 67.5%–75% of serum samples collected from 10- to 12-year-old children from various Malaysian states were seropositive.^14^ However, the relationship between SAFV infection and actual disease in humans remains equivocal, although the virus was recently reported to have been isolated from the cerebrospinal fluid of a child with aseptic meningitis.^15^

TMEV, which is a *Theilovirus* species, as is SAFV, has been shown to induce apoptosis in macrophages and necrosis in rodent cells.^16^ Apoptosis is an active process of programmed cell death that occurs as a part of normal development and aging. It can also be induced by various stimuli as an immune defense mechanism against pathogenic or noxious agents.^17^ Whether a cell dies by apoptosis depends on several conditions such as the nature of the cell death signal and the cell type.^18,19^ Previously, it was shown by Chua *et al*.^14^ that HEp-2 cells infected by SAFV-Penang exhibited cytopathic effects (CPE) suggestive of cells undergoing apoptosis. However, apoptosis induced by SAFV infection remains to be demonstrated.

There are two main mechanisms through which apoptosis is induced in cells, namely, the extrinsic and intrinsic pathways. In the extrinsic pathway, death-inducing ligands outside the cell bind to specific transmembrane death receptors and lead to the formation of a death-inducing signaling complex, which in turn activates caspase-8 and eventually caspase-3.^20^ The intrinsic pathway is initiated by changes in the inner mitochondrial membrane, which cause the opening of the mitochondrial permeability transition pore, disruption of mitochondrial transmembrane potential and release of cytochrome C into the cytosol.^21^ Cytochrome C binds and activates Apaf-1 and caspase-9 by forming an ‘apoptosome', and this eventually activates caspase-3.^22,23,24^

Understanding the interaction between a virus and cells of various provenances is crucial for the elucidation of virus infection mechanisms and pathogenesis, as well as for the prediction of the potential host range of a virus. In addition, information on the cytopathogenicity of this newly discovered human cardiovirus is grossly lacking due to the poor ability of previous clinical isolates to productively infect *in vitro* cultured cells. In this study, (i) we focus on the types of cells that are permissible to productive SAFV infection; (ii) the effect of SAFV infection on host cells; and (iii) the forms of cell death resulting from infection.

## MATERIALS AND METHODS

### Antibodies, cell lines and virus

The following antibodies used in this study were purchased commercially: rabbit anti-caspase-8 was purchased from R&D Systems (Minneapolis, MN, USA); mouse anti-caspase-9, rabbit anti-caspase-3 and rabbit anti-actin antibodies were from Cell Signaling Technology (Beverly, MA, USA); rabbit anti-mouse immunoglobulins-horseradish peroxidase and swine anti-rabbit immunoglobulins-horseradish peroxidase were from Dako (Glostrup, Denmark).

The study was performed using cell lines that were available in the laboratory and were previously obtained from American Type Culture Collection. All the cells were grown in Dulbecco's modified Eagle's medium (DMEM; Gibco, Grand Island, NY, USA) supplemented with 10% fetal bovine serum (FBS; i-DNA, Singapore) and 0.22% (w/v) sodium bicarbonate (NaHCO_3_; Sigma Aldrich, St Louis, MO, USA) and incubated at 37 °C in 5% CO_2_. The cell lines used were originally derived from human adenocarcinoma samples (HEp-2, CCL-23), African green monkey kidneys (Vero, CCL-81), mouse neuroblastoma (Neuro2A, CCL-131), mouse fibroblasts (NIH/3T3, CRL-1658), mouse kidneys (TCMK, CCL-139), mouse macrophages (J774A.1, TIB-67 and RAW 264.7, TIB-71) and hamster kidneys (CHO-K1, CCL-61).

The SAFV (SAFV-Penang strain) used in this study belongs to genotype 3 and was originally isolated in HEp-2 cells^14^ (Chua *et al.*, GenBank accession NO HQ162476.1) before it was subsequently adapted to Vero cells following eight continuous passages.

### Assay for permissible cells and kinetics of cell death

Cells were seeded overnight at a density of 1×10^6^ cells per tissue culture-treated T-25 cm^2^ flask or 5×10^4^ cells per well in 24-well plates. Virus, at a multiplicity of infection of 10, was adsorbed onto the cells for 2 h at 37 °C in 5% CO_2_ with gentle rocking every 20 min. The virus supernatant was subsequently removed, and the cells were washed twice with sterile phosphate-buffered saline (PBS) solution. Five milliliters of DMEM supplemented with 1% FBS and 0.22% NaHCO3 were ultimately added into each flask, and the inoculated cultures were incubated at 37 °C in 5% CO_2_. The inoculated cells were observed by two independent observers every 6 h. The morphology of the cells corresponding to early, mid and full CPE (approximately 10%, 50% and 100% CPE, respectively) was captured using an Olympus IX71 inverted light microscope. The times required for the respective infected cell lines to reach early, mid and full CPE were also recorded. Both the detached and adherent cells were separately harvested at various time points corresponding to the time of appearance of early, mid and full CPE. The harvested cells were pooled and pelleted by centrifugation at 300*g* for 10 min. The cell pellet was resuspended and washed twice with sterile PBS. After the last wash and centrifugation, cells were resuspended in PBS or lysis buffer depending on the downstream assay for evidence of apoptosis. The time at the point of virus inoculation into the culture medium was designated as time zero, and the experiment was repeated thrice.

### Quantitation of virus titer and virus growth kinetics assay

NIH/3T3, Neuro-2A, Vero, HEp2 and CHO-K1 cells were seeded overnight (5×10^5^ cells per well) in six-well culture plates and then infected with 10 multiplicity of infection SAFV (100 µL per well) in triplicate and incubated at 37 °C for 2 h. After 2 h of incubation, the culture supernatant in each well was removed, and the adherent cells were washed twice in sterile PBS, followed by the addition of fresh maintenance medium (DMEM, 1% FBS). The culture plates were incubated at 37 °C in 5% CO_2_. At 12-h intervals (0, 12, 24, 36, 48, 60 and 72 hours post-infection (hpi)), one plate was harvested and frozen at −80 °C. Plates were subjected to three freeze–thaw cycles, and lysates were cleared by centrifugation at 1500*g* for 5 min prior to the determination of virus titers.

Cleared supernatants were subjected to end point titration using Vero cells, and the virus titer was enumerated using the Reed and Muench method.^25^ Briefly, Vero cells (4×10^3^ cells per well) were seeded overnight in 96-well plates. Frozen supernatant containing the virus was allowed to thaw to room temperature, followed by dilution (10^−1^) in sterile 1% aqueous sodium deoxycholate (Sigma Aldrich) and vigorously mixed for 15 min to disaggregate virus. Disaggregated virus was subjected to 10-fold serial dilution in maintenance medium (DMEM, 1% FBS) and 100 µL of the diluted virus from the 10^−3^ dilution onwards was added into each well of Vero cells. Plates were incubated at 37 °C and observed daily under an inverted light microscope for the appearance of distinct CPE. Virus titer was reported as 50% cell culture-infectious doses per volume (CCID_50_/mL) and calculated using the Reed and Muench Calculator.^25^

### Positive control of apoptosis

Staurosporine (STAU; Sigma Aldrich), a fungal metabolite, used as positive control in the investigation of apoptosis induced by SAFV, is known to induce apoptotic cell death in many types of mammalian cells through both the extrinsic and intrinsic pathways. Five milliliters of DMEM supplemented with 10% FBS containing 1×10^6^ cells of the respective permissible cell lines were added to tissue culture-treated T-25 cm^2^ flasks. After overnight incubation, 1 µM of staurosporine was added to the culture media, and both the detached and adherent cells were separately harvested following another 4 h of incubation. The harvested cells were pooled together and pelleted by centrifugation at 300*g* for 10 min. The cell pellet was resuspended and washed twice with sterile PBS. After the last wash and centrifugation, cells were resuspended in PBS or lysis buffer, depending on the downstream application used to determine the presence of apoptosis.

### DNA laddering assay

The pooled SAFV-infected or staurosporine-treated cells were separately washed with PBS and treated with cell lysis buffer (1% NP-40 in 20 mM EDTA, 50 mM Tris-HCl, pH 7.5) for 10 s. The supernatant was collected following centrifugation, supplemented with 1% sodium dodecyl sulfate (SDS) and treated with 5 µg/µL of RNase A for 2 h at 56 °C. Subsequently, samples were incubated in 2.5 µg/µL of proteinase K for 2 h at 37 °C, and then supplemented with 0.5 volume of 10 M ammonium acetate and 2.5 volumes of ice-cold ethanol. Suspensions were incubated at −80 °C for 1 h and centrifuged for 20 min at 12 000 rpm. The pellet was washed with 80% ice-cold ethanol, air-dried at room temperature for 10 min and dissolved in Tris-HCl/EDTA (TE) buffer. The quantity of DNA was measured via spectrophotometry and 4 µg of DNA was loaded into wells of 2% agarose gel for electrophoresis run.^26^ The experiments involving the DNA laddering assay were repeated independently three times.

### Apo-ONE fluorometric assay

Only the infected cells harvested at mid CPE were used in the Apo-ONE fluorometric assay to determine the presence of SAFV-induced apoptosis. Both infected and mock-infected cell monolayers were harvested, as described earlier, and treated with RIPA buffer (0.5% NP-40, 0.5% deoxycholic sodium, 0.005% SDS in 50 mM Tris, pH 8.0, 250 mM NaCl) and Complete Protease Inhibitor (Roche Applied Science, Singapore). The treated cells were then subjected to five freeze–thaw cycles. Apoptotic activity due to caspase-3/7 activation was subsequently measured using an Apo-ONE Fluorometric Assay Kit (Promega Corporation, Madison, WI, USA) according to the manufacturer's protocol.

### Western blot

The pooled harvested SAFV-infected or staurosporine-treated cells were washed with PBS and treated with Complete Lysis-M and Complete Protease Inhibitor (Roche Applied Science) for 5 min. Protein samples (20 µg each) were electrophoresed in 16% SDS polyacrylamide gels and transferred onto polyvinylidene difluoride membranes (Bio-Rad, Philadelphia, PA, USA). Membranes were blocked for 1 h at room temperature in a suspension of 5% (w/v) blotting grade non-fat milk dissolved in PBS supplemented with 1% Tween-20 (PBS-T). Membranes were incubated overnight at 4 °C with the primary antibody in PBS-T buffer supplemented with 5% non-fat milk. The membranes were washed three times and subsequently incubated at room temperature for 1 h with anti-mouse or anti-rabbit immunoglobulins-horseradish peroxidase in 5% (w/v) non-fat milk in PBS-T. The Western blotting experiments were independently repeated three times.

### Statistical analysis

A paired Student's *t*-test was used in this study and differences were considered statistically significant at *P*<0.05 and extremely significant at *P*<0.01.

## RESULTS

### SAFV is able to infect primate and rodent cell lines and the kinetics of cell death vary

**Figure 1:**
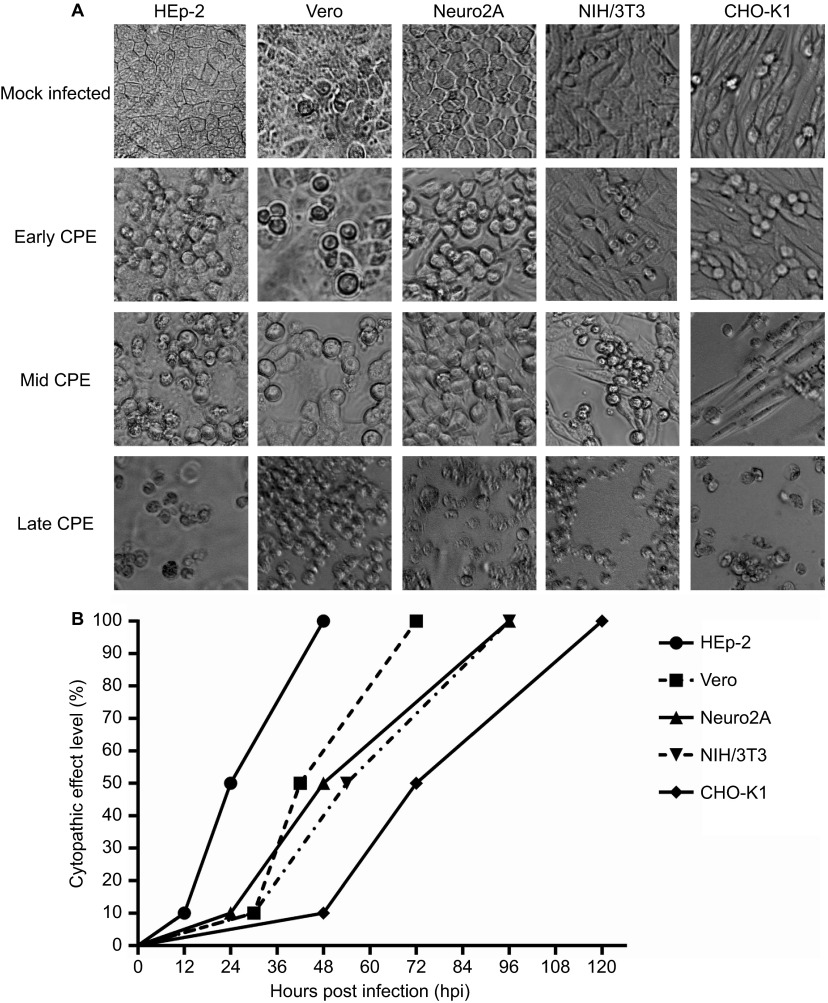
(**A**) Morphology of the early, mid and full CPEs in SAFV-infected cell lines. Mock-infected cells served as controls for the normal cellular morphology of the respective cell lines. Magnification: ×200. (**B**) Kinetics of cell death following infection by SAFV. The graph shows the times required for SAFV-infected HEp-2, Vero, Neuro2A, NIH/3T3 and CHO-K1 cells to attain early (10%), mid (50%) and full (100%) CPEs. The CPE levels of the infected cell lines were assessed by two independent researchers every 6 h following infection until the cultured cells achieved full CPE. The three points on the graph correspond to the time points at which the SAFV-infected cell monolayer attained early, mid and full CPE.

Of the eight cell lines infected with laboratory-adapted SAFV, all except TCMK, J774A.1 and RAW 264.7 (mouse cell lines) were permissible to productive SAFV infection. The representative patterns of early, mid and full CPE in the permissible cell lines are shown in Figure 1A. The infected cell lines exhibited varying degrees of CPE at different time points and generally, cell lines of human origin such as HEp-2 exhibited earlier evidence CPE and a more rapid rate of progression to reach full CPE; by contrast, the slowest rate of CPE progression was observed in cells of hamster origin—CHO-K1 (Figure 1B). The first evidence of cell death (CPE) was observed in HEp-2 cells at 12 hpi, while initial evidence of CPE in CHO-K1 cells was observed at 48 hpi. The sequential order in which the five cell lines infected with the same inoculum of SAFV showing early (10%) CPE was as follows: HEp-2, Neuro2A, Vero and NIH/3T3, and CHO-K1. The appearance of 100% CPE was observed in HEp-2 at 48 hpi, whereas full cell death was observed at 120 hpi in CHO-K1 cells. Progression to full CPE was also relatively rapid in monkey Vero cells (42 h) when compared to the mouse NIH/3T3 (54 h) cell line. The sequence in which the five infected cell lines attained full CPE was in the following order: HEp-2, Vero, Neuro2A and NIH/3T3, and CHO-K1. Neuro2A cells, quite surprisingly, exhibited CPE early after infection (24 hpi) and had achieved full CPE at 96 hpi. All cell lines showed 10% CPE between 12 and 48 hpi, 50% CPE between 24 and 72 hpi and 100% CPE between 48 and 120 hpi. Thus, of the cell lines tested for infection, SAFV was able to infect HEp-2, Vero, Neuro2A, NIH/3T3 and CHO-K1 and caused different degrees of cell death at various time points. The SAFV antigen present within infected cells that had undergone CPE was confirmed by indirect immunofluorescence assay using commercial cross-reacting pan-enterovirus monoclonal antibody (Merck Millipore, Billerica, MA, USA) and anti-SAFV VP1 protein specific rabbit's polyclonal antibodies prepared in the laboratory. Purified SAFV VP1 proteins for immunizing rabbits were produced by recombinant DNA technology using bacterial (*Escherichia coli* M15) protein expression system and plasmid vector pQE30 (immunofluorescence assay-positive photographs and rabbit anti-SAFV VP1 polyclonal antibodies are available upon request).

### SAFV produces a high virus titer in infected cells

**Figure 2:**
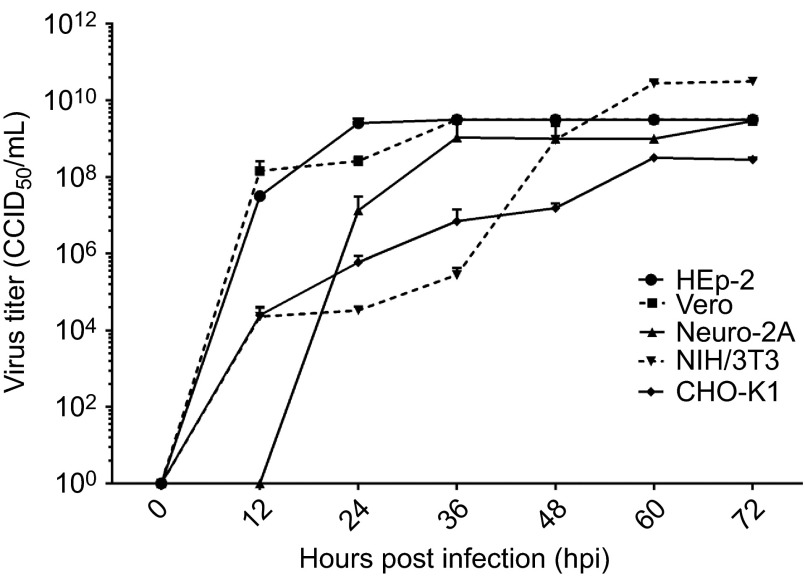
Kinetics of virus growth in SAFV-infected cell lines. Virus titers in culture supernatants expressed as lgCCID_50_/mL were determined at various 12-h intervals (0, 12, 24, 36, 48, 60 and 72 hpi). Virus titers in culture supernatants were quantified by microneutralization assays using Vero cells. The end point virus titer was enumerated according to the Reed and Muench method ^25^ and is reported as the CCID_50_. The experiments were performed thrice and the average values with standard deviations are plotted. CCID_50_, 50% cell culture infectious dose.

The culture supernatants harvested from infected cells were titrated to assess the productivity of SAFV in these cell lines (Figure 2). Virus titers of culture supernatants obtained from infected cells reached a detectable level by 12 hpi for HEp-2, Vero, NIH/3T3 and CHO-K1 and by 24 hpi for Neuro2A. At the time points that virus titers were detectable, Vero cells attained the highest titer (1.45×10^8^ CCID_50_/mL, 8.16 lgCCID_50_/mL), followed by HEp-2 (3.16×10^7^ CCID_50_/mL, 7.50 lgCCID_50_/mL), Neuro2A (1.33×10^7^ CCID_50_/mL, 7.12 lgCCID_50_/mL), CHO-K1 (2.46×10^4^ CCID_50_/mL, 4.39 lgCCID_50_/mL) and NIH/3T3 (2.26×10^4^ CCID_50_/mL, 4.35 lgCCID_50_/mL). Murine NIH/3T3 cells unexpectedly supported efficient virus production, with virus titers of 3.16×10^10^ CCID_50_/mL and 10.50 lgCCID_50_/mL at 72 hpi. At the same time point, other cell lines infected by SAFV also attained high titers: 3.16×10^9^ CCID_50_/mL and 9.50 lgCCID_50_ mL for HEp-2 and Vero, 2.90×10^10^ CCID_50_/mL and 9.46 lgCCID_50_/mL for Neuro2A, and 2.82×10^8^ CCID_50_/mL and 8.45 lgCCID_50_/mL for CHO-K1. In summary, SAFV can achieve high virus titers following infection in the cells tested.

### DNA laddering was observed in all SAFV-infected cells except HEp-2

**Figure 3:**
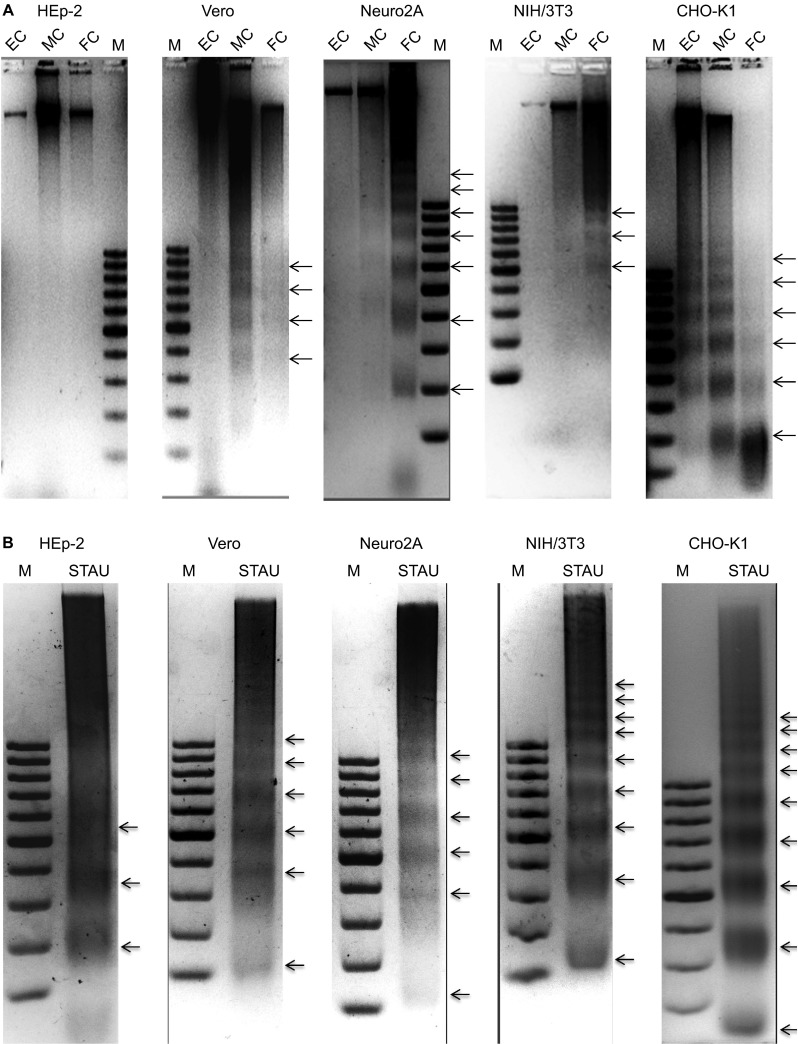
DNA laddering analysis to demonstrate end-stage apoptotic cell death. (**A**) DNA laddering analysis of Saffold virus-infected cell lines at the early, mid and full CPE time points. The Vero, Neuro2A and CHO-K1 cell lines exhibited DNA ‘smearing' patterns with early CPE. The NIH/3T3 and HEp-2 cell lines only showed such patterns from mid CPE onward. The CHO-K1 cell line exhibited the characteristic DNA laddering pattern with early CPE. The Neuro2A and Vero cell lines showed the DNA laddering pattern beginning at mid CPE and the NIH/3T3 cell line only showed this pattern at full CPE. Infected HEp-2 cells did not show a DNA laddering pattern. Lanes EC, MC and FC, represent DNA extracted from the SAFV-infected cells showing early, mid and full CPE, respectively. Lanes marked ‘M' denote the DNA molecular size marker. (**B**) DNA laddering analysis of STAU-treated cells as positive controls. All cell lines showed a DNA laddering pattern when harvested at 4 h following STAU treatment. Lanes marked ‘STAU' represent DNA extracted after staurosporine treatment. Lanes marked ‘M' denote the DNA molecular size marker. The experiments were performed in triplicate and one of the three results is presented.

To determine the forms of cell death exhibited by the cell lines following infection with SAFV, DNA was extracted from infected cells at different time points corresponding to early, mid or full CPE, and DNA laddering assays were performed (Figure 3A). All infected cell lines exhibited DNA ‘smearing' beginning from either the early or mid CPE time points. Distinct DNA ‘ladder' patterns were also observed in all the cell lines except HEp-2. To exclude the possibility that the cell lines, especially HEp-2 cells, could not undergo end-stage apoptosis and thus show the ‘laddering' pattern, the DNA of each of the respective permissible cell lines was extracted following treatment with STAU, and the DNA laddering assay was performed as described above (Figure 3B). The results showed that all the cell lines treated with STAU, including HEp-2 cells, were able to undergo the end-stage apoptosis hallmark of DNA fragmentation by showing a DNA ‘laddering' pattern. These results suggest that the infected Vero, Neuro2A, NIH/3T3 and CHO-K1 cells underwent end-stage apoptosis, whereas in SAFV-infected HEp-2 cells, apoptosis did not proceed to the stage of DNA fragmentation.

### All the SAFV-infected cells exhibited significant caspase-3/7 activity at mid CPE

**Figure 4:**
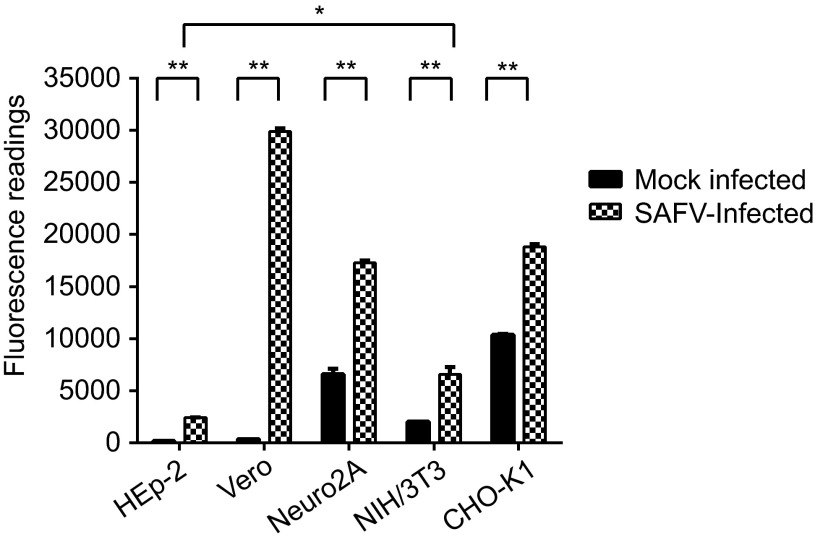
Apo-ONE Fluorometric assay of SAFV-infected HEp-2, Vero, Neuro2A, NIH/3T3 and CHO-K1 cell lines harvested at mid CPE in comparison with mock-infected cells. A paired Student's *t*-test was used to compare the fluorometric readings, and differences were considered significant at *P<0.05 and extremely significant at **P<0.01. All experiments were performed in triplicate and the average values with standard deviations are plotted.

To confirm the apoptotic activity detected in the DNA laddering assay, cell lysates of the infected and mock-infected cells harvested at mid CPE were used to detect the activation of caspase-3 or caspase-7 using the Apo-ONE fluorometric assay. All the infected cells lines exhibited significant increases in the levels of caspase-3 or caspase-7 activities in comparison to mock-infected cells (*P*<0.01) upon reaching mid CPE (Figure 4). Vero cells exhibited the highest fluorescence reading among the five infected cell lines, followed by CHO-K1, Neuro2A, NIH/3T3 and lastly HEp-2. The fluorescence reading of HEp-2 cells was the lowest among the five cell lines and was significantly lower than the fluorescence reading of the NIH/3T3 cell line, which had the next to lowest reading among the infected cell lines (*P*<0.05).

### SAFV induced apoptosis through both the extrinsic and intrinsic pathways

**Figure 5:**
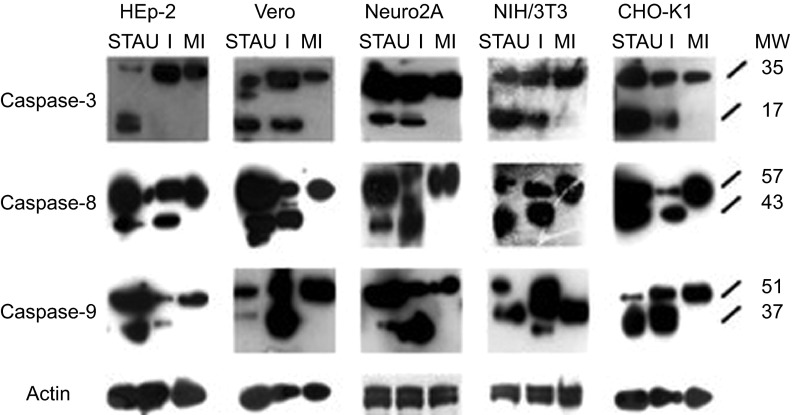
Western blots of cell lysates derived from SAFV-infected HEp-2, Vero, Neuro2A, NIH/3T3 and CHO-K1 cell lines harvested at mid CPE in comparison with respective mock-infected control and STAU-treated control cells. Membranes were stained with respective antibodies against caspases-3, -8, -9 and actin. The intensity of actin staining served as the protein loading control. Lanes with I denote infected cell lysates, lanes with MI denote mock-infected control cell lysates, lanes with STAU denote STAU-treated control cell lysates and lanes with MW indicate the protein molecular mass in kDa. Caspases-3, -8 and -9 were cleaved to their respective active forms (p17, p43 and p37) in SAFV-infected Vero, Neuro2A, NIH/3T3, CHO-K1 cells and all STAU-treated cells. Only caspases-8 and -9 were cleaved to their active forms in SAFV-infected HEp-2 cells. The Western blotting results shown here are representative one of the three replicate experiments.

To further confirm the apoptotic activity of the SAFV-infected cells and to determine the apoptotic pathway involved, total proteins from STAU-treated, mock-infected and SAFV-infected cell lysates harvested at mid CPE were subjected to polyacrylamide gel electrophoresis and Western blotting. Membranes were stained with the respective antibodies against caspase-3, -8 and -9 and actin. The intensity of actin staining served as protein-loading control to ensure that a near-equal amount of cell lysate from each respective cell line was loaded into each well. The cleavage of procaspases-8 and -9 into their activated molecular mass 43 and 37 kDa forms, respectively, was detected in both STAU-treated and SAFV-infected cell lysates derived from all cell lines and absent in the mock-infected cell lysates (Figure 5). Cleavage of procaspase-3 into the 17 kDa active form was also detected in cell lysates derived from all the STAU-treated and SAFV-infected cell lines, except in SAFV-infected HEp-2 cells (Figure 5). SAFV induced apoptosis through both the extrinsic and intrinsic pathways, but in HEp-2 cells, apoptosis did not proceed to the stage of caspase-3 cleavage.

## DISCUSSION

Following the discovery of SAFV, several research groups have attempted to adapt the virus to grow in various cell lines to study the facets of its growth kinetics and pathogenesis. Such cells include WI-38 (lung-derived human embryonic fibroblasts), HFDK (human fetal diploid kidneys), A-549 (human lung adenocarcinoma epithelia), BSC (African green monkey renal epithelia) and RD (human rhabdomyosarcoma) cells,^5^ but the virus could not be recovered from these cells. Zoll *et al.*^3^ developed a method to grow an SAFV-3 isolate from clinical specimens in cultured cells and performed further studies on the virus as able within the limitations of this method. Similarly, SAFV-Penang, also belonging to genotype 3, was first isolated in HEp-2 cells and was shown to productively infect a variety of cell lines of primate and rodent origins following adaptation in Vero cells. Identification of cells that support productive SAFV infection provides an indication of the virus's potential host range and allows phenotypic characterization of infected cells in *vis-à-vis* host–pathogen interactions. However, because SAFV is recalcitrant to replicate in most cell cultures according to previous reports, these aspects of virus host cells interactions are not well studied. Our aim was to provide a platform for further studies of host cell–SAFV interactions by characterizing some cell lines that could be productively infected.

Eight cell lines from American Type Culture Collection were inoculated with SAFV-Penang to determine their potential to support productive virus growth. Full CPE, as characterized by 100% lysis of the cell monolayer, was observed in all infected cells with the exception of the murine kidney (TCMK, CCL-139) and mouse macrophage (J774A.1, TIB-67 and RAW 264.7, TIB-71) cell lines. Infected cells of human origin such as HEp-2 exhibited rapid cell death, as full CPE was achieved within 48 hpi. It was also observed to support efficient virus replication, with the virus titer reaching 3.16×10^9^ CCID_50_/mL. Vero cells of monkey origin also supported efficient virus replication and underwent rapid CPE upon infection. The infected cells exhibited full CPE within 72 hpi and attained a virus titer of 3.16×10^9^ CCID_50_/mL, which was as high as that of HEp-2. In this study, SAFV was also found to be able to productively infect rodent cells, although less efficiently. This suggests that the virus has the potential to infect other animals, specifically rodents. A preliminary study in our laboratory, which was approved by the institutional ethics committee, demonstrated that SAFV-Penang was able to cause sickness in BALB/c suckling mice following intracerebral inoculation (unpublished data). The mouse neuronal cell line, Neuro2A, supported viral replication to nearly the same extent as human cells, as shown by the fairly similar virus titers at full CPE. However, the differences in the ability of the virus to induce CPE in cells, as well as the variation in replication rates, resulting in differing virus titers, remain unclear, although a recently published study by Hemida *et al*.^27^ demonstrated that variation in virus pathogenesis may be influenced by the expression of receptor(s) on the host cells.

A key aspect of the interaction of SAFV with the host cells is its ability to induce full lysis of the infected cell monolayer. It was therefore prudent to determine what type of cell death is induced by SAFV in infected cells. TMEV, a murine cardiovirus that is phylogenetically closely related to SAFV, was previously shown to induce both apoptosis and necrosis in cultured cells.^16^ Thus, we explored the ability of SAFV to induce both apoptosis and necrosis in infected cell monolayers. DNA fragmentation is a key feature of end-stage apoptosis and can be visualized through DNA laddering assays. All infected cells, with the exception of HEp-2, exhibited both DNA smearing and laddering indicative of necrotic and apoptotic cell death, respectively. HEp-2 exhibited only smearing even later into the infection cycle, implying the absence of end-stage apoptotic cell death. Moreover, caspase-3 cleavage was also observed in all cell lines except HEp-2 (Figure 5). The presence of apoptotic activity induced by SAFV infection was confirmed by the Apo-ONE fluorometric assay. The levels of caspase-3/7 activation in all cell lines tested were extremely significantly higher compared to mock-infected controls, while the levels of caspase-3/7 activation in HEp-2 were the lowest, which might explain the absence of DNA laddering and caspase-3 cleavage noted in infected HEp-2 cells at mid CPE (Figures 3 and 5). This finding suggests that the caspase-3 activation in HEp-2 cells could have been reduced or blocked by an unknown protein, thus preventing the cleavage product from reaching a sufficient level to subsequently cause the cells to undergo end-stage apoptosis.

There are two main mechanisms through which apoptosis is induced in cells, namely, the extrinsic and intrinsic pathways. The Western blot data showed the cleavage of procaspase-8 and procaspase-9 into their respective molecular mass 43 and 37 kDa forms in all cell lines infected by SAFV-Penang, implying that activation of apoptosis occurred through both the intrinsic and extrinsic pathways. Procaspase-3 is the final executor in the apoptotic signaling cascade and is responsible for chromatin cleavage and DNA laddering. Its cleavage into its active molecular mass 17 kDa form was also observed in all cell lines except HEp-2. The infection of HEp-2 cells with SAFV-Penang led to the activation of both the intrinsic and extrinsic pathways, but failed to progress further into the full activation of caspase-3 and resultant DNA laddering. The molecule(s) responsible for blocking the progression into the late stages of apoptosis in HEp-2 cells are not known and are under investigation in our laboratory. Recently, Fanet *et al.*^28^ determined that transfection of the TMEV Leader protein into BHK-21 cells resulted in the induction of apoptosis between 24 and 48 h post-transfection via the intrinsic pathway.^28^ Whether SAFV Leader peptide can induce apoptosis in a manner similar to the L protein of TMEV would be an interesting subject of inquiry and is also currently under investigation in our laboratory. Further characterization of the key host cell and viral proteins involved in the dynamics of host cell–pathogen interactions and that have either pro-apoptotic or anti-apoptotic activities in response to SAFV infection would also be an interesting area of investigation.

In conclusion, SAFV-Penang can infect a number of primate and rodent cell lines, including HEp-2, Vero, Neuro2A, NIH/3T3 and CHO-K1, and induce the host cells to undergo apoptosis through both the extrinsic and intrinsic pathways. However, the late stage apoptotic activities of HEp-2 cells are blocked. In future studies, we will focus on determining the viral protein(s) causing apoptosis and the mechanism underlying the apoptosis inhibition in HEp-2 cells.
